# The Role of Body Weight on Bone in Anorexia Nervosa: A HR-pQCT Study

**DOI:** 10.1007/s00223-017-0254-7

**Published:** 2017-02-21

**Authors:** Jacob Frølich, Stinus Hansen, Laura Al-Dakhiel Winkler, Andreas K. Andresen, Anne Pernille Hermann, René K. Støving

**Affiliations:** 1 Centre for Eating Disorders, Department of Endocrinology, Odense University Hospital & Psychiatry of Region Southern Denmark, University of Southern Denmark, Odense, Denmark; 2 Department of Endocrinology, Odense University Hospital, Odense, Denmark; 3 Center for Spine Surgery and Research, Region of Southern Denmark, Middelfart, Denmark; 4 Centre of Eating Disorders and Department of Endocrinology, Odense University Hospital, Kloevervaenget 10, 6th floor, 5000 Odense C, Denmark

**Keywords:** Anorexia nervosa, High-resolution peripheral quantitative computed tomography, Bone microarchitecture, Bone geometry, Mechanical loading

## Abstract

Anorexia nervosa (AN) is associated with decreased bone mineral density and increased risk of fracture. The aim of this study was to assess bone geometry, volumetric bone mineral density (vBMD), trabecular microarchitecture and estimated failure load in weight-bearing vs. non-weight-bearing bones in AN. We included twenty-five females with AN, and twenty-five female controls matched on age and height. Bone geometry, vBMD and trabecular microarchitecture were assessed using high-resolution peripheral quantitative computed tomography of the distal radius and tibia. At both sites, cortical perimeter and total bone area were similar in patients and controls. Total vBMD was lower in the AN group in the tibia (*p* < 0.0005) but not in the radius. In the tibia, cortical thickness was approximately 25% lower (p < 0.0005) in the AN group, whereas there was no significant difference in the radius. In terms of trabecular microarchitecture, all indices [bone volume/tissue volume (BV/TV); trabecular thickness (Tb.Th.), trabecular number (Tb.N) and trabecular spacing (Tb.Sp.)] were impaired in AN in the tibia (*p* values range < 0.01–0.0001). In the radius, BV/TV and Tb.N were lower (*p* < 0.05 and *p* < 0.001, respectively); Tb.Sp. was higher (*p* < 0.001), whereas Tb.Th. did not differ, compared to controls. Estimated failure load was lower in patients in both the radius and the tibia (*p* < 0.0005 and *p* < 0.0001, respectively), most pronounced in the tibia. In conclusion, the impairment of cortical thickness and estimated failure load were significantly more pronounced in the weight-bearing tibia, compared to the non-weight-bearing radius, implying a direct effect of low body weight on bone loss in AN.

**Electronic supplementary material:**

The online version of this article (doi:10.1007/s00223-017-0254-7) contains supplementary material, which is available to authorized users.

## Introduction

Anorexia nervosa (AN) is a psychiatric disorder affecting up to 2% of young women in the Western world [1]. The disease is characterized by low body weight due to self-imposed weight loss (or resistance to weight gain), as well as a distorted self-image. The concomitant malnutrition is associated with several endocrine disturbances including hypothalamic hypogonadism, hypercortisolemia and resistance to growth hormone, each of which is known to have deleterious effects in bone [2]. Accordingly, patients with AN have been shown to have decreased bone mineral density (BMD) [3], and impaired bone microarchitecture, even with short duration of disease [4, 5]. What is of particular concern is the fact that the onset of AN often coincide with puberty [6], a critical period for the accrual of peak bone mass (PBM) [7], a known predictor for the development of osteoporosis [8]. Accordingly, a Danish register-based study found patients with AN to have an almost twofold relative risk for overall fracture and a sevenfold relative risk for hip fractures, compared to the general population [9]. The mechanism behind bone loss in AN is undoubtedly multifactorial, and the abovementioned endocrine disturbances have been established as important factors. However, the role of body weight per se, and the mechanical load on the skeleton, is yet to be determined. It is well known that the skeleton has potential to adjust to changes in load, presumably with the osteocytes as the primary mechanosensing bone cells. When mechanical load increases, the secretion of sclerostin from osteocytes decreases, ultimately causing new bone formation by the osteoblast by stimulating the wnt/B-catenin signalling pathway [10]. In accordance, patients with AN have higher levels of sclerostin, lower levels of bone formation markers and higher levels of bone resorption markers, compared to controls [11]. One way to assess the effect of mechanical loading in vivo is to compare bone characteristics in weight-bearing vs. non-weight-bearing bones. To the best of our knowledge, such a comparison is yet to be made in patients with AN. When applying a similar approach to overweight patients referred to bariatric surgery, we have recently shown that obesity favours increased cortical area and cortical thickness (despite no significant difference in total bone area) in the tibia compared with normal weight, height-matched controls. The same pattern did not apply to the radius [12].

One major issue regarding the assessment of bone loss in AN is that areal BMD (aBMD) measurement is based on a 2-dimensional image, (dual-energy X-ray absorptiometry, DXA) which to a certain degree fails to take weight loss-induced changes in extra-osseous fat distribution and bone marrow adiposity, into account [13]. The consequence is that BMD may be underestimated in the nadir-weight state, and overestimated upon subsequent weight-gain [13]. The introduction of high-resolution peripheral quantitative computed tomography (HR-pQCT) allows for 3-dimensional (volumetric) assessment of BMD (vBMD), as well as compartment specific geometry and bone microarchitecture. Although only assessed for quantitative computed tomography (QCT), the 3-dimensional technique seems to minimize the influence of fat distribution on BMD assessment [14].

In this cross-sectional study, we therefore used both DXA and HR-pQCT to compare bone characteristics in patients with AN, with age- and height-matched control subjects. We hypothesized that aBMD, vBMD and bone microarchitecture would be significantly impaired in AN patients, compared to controls at all bone sites. We further hypothesized that this impairment would be more pronounced in weight-bearing compared to non-weight-bearing bones.

## Subjects and Methods

### Study Subjects

Patients were recruited from Centre for Eating Disorders, Odense University Hospital. Upon referral to the centre, all patients were diagnosed by a trained physician according to the ICD-10 diagnostic criteria for AN (body weight <85% of expected, self-induced weight-loss, distorted body image and amenorrhea). Inclusion criteria were female gender, Caucasian ethnicity, and age between 18 and 40 years. In order to reflect the population of patients with AN, and to ensure external validity, a minimum duration of disease of three years was chosen. Exclusion criteria were other chronic diseases known to affect bone or medication affecting bone metabolism. Estrogen-containing oral contraceptive pills (OCPs) were allowed.

Each AN case was matched with a control subject on height (±3 cm) and gender. Potential control subjects were randomly identified from a sample from the Danish Civil Registration Registry combining date of birth, gender and residence in the Municipality of Odense, Denmark. When responding to a written invitation to participate, potential controls declared their height to allow for correct matching. Inclusion criteria for controls, in addition to matching criteria, were normal weight according to the WHO definition [15], and age within the same age-range as the patients (18–40 years). Exclusion criteria were amenorrhea (no menstrual bleeding for three or more consecutive months) of any cause, a history of any eating disorder and diseases known to affect bone or medications as stated above.

Prior to inclusion, we performed sample size calculation, based on the data provided by Ackerman and colleagues [16]. Based on trabecular number suggested inclusion of 25 patients and 25 controls allows for an alpha of 0.05 and a power of 80%.

### DXA

DXA (Hologic, Discovery, Waltham, MA, US) of the hip and lumbar spine was used to assess bone mineral content (BMC) and aBMD at the femoral neck, trochanter region, total hip and lumbar spine (L1–L4). A whole body DXA scan was performed to obtain indices of body composition, including total fat mass and fat percentage. The coefficient of variation (CV) for hip and spine assessment is 1.5% in our unit.

### HR-pQCT

Values for bone geometry, compartment-specific vBMD and bone microarchitecture were obtained using a high-resolution peripheral quantitative computed tomography system (HR-pQCT, XtremeCT; Scanco Medical, Zürich, Switzerland). Validation, correlation to μCT measurements and methods for parameter extraction from the images, has previously been reported by Laib et al. [17–19].

We applied the manufactures’ default protocol for in vivo imaging, which has been described in detail elsewhere [20]. Images of the non-dominant distal radius and the tibia were obtained, except in case of previous fracture, where the non-fractured limb was chosen. The subject’s forearm/ankle was immobilized in a carbon fibre cast to decrease motion artefacts. Based on an initial 2D scout scan, the operator placed a reference line at the bone endplate and the region of interest initiated 9.5 mm and 22.5 mm from the radius and the tibia endplate, respectively, and extended 9.02 mm in the proximal direction. Each region contained 110 parallel slices with an 82-μm isotropic voxel size.

The operator immediately viewed the images for visual motion-artefacts, and up to two additional scans of each region was performed to secure the best possible quality of the scan.

Parameter extraction was performed in accordance with the manufactures protocol. In brief, scans were automatically segmented into trabecular and cortical compartments, using a threshold-based algorithm [17], thereby providing information on cortical area, trabecular area, cortical thickness (Ct.Th.) and compartment specific volumetric densities. Trabecular bone volume per tissue volume (BV/TV) was derived from trabecular vBMD under the assumption of fully mineralized bone having a mineral density of 1200 mg hydroxyapatite per cm^3^. Assessment of trabecular number (Tb.N.) was based on direct determination of ridge number density and the principle of 3D distance transformation, as described by Laib et al. [18]. From BV/TV and Tb.N., trabecular thickness (Tb.Th.) and trabecular spacing (Tb.Sp.) was calculated as Tb.Th. = (BV/TV)/Tb.N. and Tb.Sp. = (1 − BV/TV)/Tb.N, respectively.

Assessment of cortical porosity (Co.Po.) was based on extended cortical evaluation, as described by Burghardt et al. [21], and calculated as void cortical volume divided by total cortical volume. Finally, estimated bone strength was calculated by Finite Element Analysis (FEA) software, provided by the manufacturer (μFE Element Analysis Solver v.1.15; Scanco Medical, Brüttisellen, Switzerland). In the FEA, an axial compression test was simulated, wherein failure load was defined as the force that will result in more than 2% of elements being strained beyond 0.7%, thereby simulating fracture. Also bone stiffness was reported as an estimate of bone strength.

In our unit, CV ranged 0.4–0.8% for densities, 3.5–5.0% for trabecular microarchitecture parameters, 1.0–7.2% for extended cortical measures, and 1.2–1.7% for FE estimated failure load [22].

### Blood Samples

Blood samples from patients and controls were analysed for ionized calcium, 25-hydroxy vitamin D and parathyroid hormone (PTH). In order to rule out exclusion criteria and potential competing reasons for amenorrhea (premature ovarian failure or hyperprolactinemia), blood samples were further analysed for thyroid-stimulating hormone (TSH), alkaline phosphatase (ALP), luteinizing hormone (LH), follicle-stimulating hormone (FSH) and prolactin.

### Questionnaire

A questionnaire provided information on age at diagnosis of AN and duration of disease (patients) as well as information on history of fracture, family history of osteoporosis, medical history, medication, alcohol and smoking habits (all participants).

### Height and Weight

Height was measured on a wall-mounted stadiometer, and weight was measured on a calibrated platform scale. BMI was calculated as weight divided by the square of height (kg/m^2^).

### Statistics

Normality of data was evaluated mathematically by the Shapiro–Wilk test and visually by normal probability plots. Non-normally distributed parameters were further evaluated with histograms to determine distribution. Data are presented as mean +/− SD for normally distributed data or median and interquartile range for non-normally distributed data. Differences between groups were assessed using t-test, Wilcoxon–Mann–Whitney test or Moods median test, as appropriate. For evaluation of categorical outcomes, a Chi-squared test was used. As any potential effect of OCPs on bone microarchitecture in AN have not been studied, sensitivity analysis were required to assess the potential impact of OCP use on the HR-pQCT-derived parameters. Specifically, we performed multiple regression analysis, in order to adjust any potential effect of OCP use for the impact of BMI and duration of disease.

To estimate the association between bone measures and body weight, we performed seemingly unrelated regression (SUR) modelling [23]. In the regression, we assessed the difference in selected variables between patient and control in each height-matched pair versus the difference in weight in the same pair. Thus, the regressions describe the predicted change in each variable when the patients weight decreases compared to controls. The difference between weight-bearing and non-weight-bearing bone, were then evaluated with a post estimation Wald test. All values in the regression analysis were adjusted for smoking.

A *p* value < 0.05 was considered significant. Stata (Version 14.0, StataCorp, TX, US) was used for all statistical analysis.

## Results

### Study Subjects

A total of 28 patients were included in the study. Three patients were excluded before matching due to osteomalacia (*n* = 1), use of glucocorticoids (*n* = 1) and a height too low to obtain a match (153 cm, *n* = 1), as we did not consider a randomly drawn control of similar height, representative of the general population. No other subjects were identified with other diseases known to affect bone, or competing reasons for amenorrhea. Thus, a total of 25 patients were eligible for matching.

Subject characteristics are shown in Table 1. Patients and controls did not differ in terms of age or height (due to matching), but as intended, in terms of weight, as well as body composition (fat percentage, fat mass and lean body mass). Controls had a younger mean age at menarche, compared to AN cases (*p* < 0.05). Levels of ionized calcium, PTH and 25-hydroxy-vitamin D were not different between groups, nor were the number of subjects with values of 25-hydroxy-vitamin D below reference range (six subjects in each group, data not shown). A higher prevalence of smoking as well as a history of at least one fracture was found in the patient group (*p* < 0.05 for both). The prevalence of OCP use was equal between groups.

**Table 1:** Subject characteristics

Characteristics	AN (*n* = 25)	Control (*n* = 25)	*p* value
Age (years)	27.5 (23.8; 29.6)	27.9 (23.8; 31.4)	0.68
Height (cm)	166.6 ± 6.0	167.3 ± 6.0	0.71
Weight (kg)	44.8 ± 4.9	66.0 ± 9.2	<**0.0001**
BMI (kg/m^2^)	16.2 ± 1.25	22.8 ± 2.7	<**0.0001**
Age at menarche	12.9 ± 1.39	11.8 ± 0.89	<**0.05**
Duration of disease (years)	7.8 ± 4.4	–	
Age at onset of AN (years)	20.0 (15.0; 22.0)	–	
Body composition			
Fat mass (kg)	9.0 ± 3.4	21.8 ± 7.0	<**0.0001**
Fat percentage (%)	19.9 ± 6.4	31.8 ± 6.8	< **0.0001**
Lean mass (kg)	34.4 ± 3.6	42.3 ± 3.7	< **0.0001**
Blood samples			
Ca^2+^ (mmol/L)	1.25 ± 0.05	1.23 ± 0.03	0.07
25-OH vitamin D (nmol/L)	83 ± 47	70 ± 27	0.22
PTH (pmol/L)	3.6 (1.8; 5.6)	3.9 (2.6; 5.0)	0.33
Current OCP use (yes/total)	9/25	7/25	0.54
Current smoker (yes/total)	12/25	4/25	<**0.05**
History of at least one fracture (yes/total)	11/25	3/25	<**0.05**

Values are presented as mean ± SD or median and interquartile range, as appropriate

*P* values in bold indicates *p* < 0.05 on difference between groups

*BMI* body mass index, *AN* anorexia nervosa, *PTH* parathyroid hormone, *OCP* oral contraceptive pill

### DXA

BMC and BMD were significantly lower in patients compared to controls, at all sites (*p* < 0.0001) (Table 2).

**Table 2:** DXA measurements

	AN (*n* = 25)	Controls (*n* = 25)	*p* value
Femoral neck BMC (g)	3.04 ± 0.49	4.05 ± 0.62	<**0.0001**
Femoral neck BMD (g/cm^2^)	0.60 ± 0.10	0.79 ± 0.10	<**0.0001**
Trochanteric BMC (g)	5.31 ± 0.88	7.29 ± 1.15	<**0.0001**
Trochanteric BMD (g/cm^2^)	0.50 ± 0.07	0.68 ± 0.08	<**0.0001**
Total hip BMC (g)	23.3 ± 3.87	31.3 ± 4.65	<**0.0001**
Total hip BMD (g/cm^2^)	0.68 ± 0.11	0.91 ± 0.09	<**0.0001**
Total spine (L1–L4) BMC (g)	46.00 ± 10.2	59.1 ± 9.5	<**0.0001**
Total spine (L1–L4) BMD (g/cm^2^)	0.79 ± 0.13	0.99 ± 0.10	<**0.0001**

Values are presented as mean ± SD. P-values in bold indicates *p* < 0.05 on difference between groups

*BMC* bone mineral content, *BMD* bone mineral density

### HR-pQCT

HR-pQCT results are shown in Table 3.

**Table 3:** HR-pQCT measures in patients and controls

Geometry	Radius			Tibia		
	AN (*n* = 25)	Controls (*n* = 25)	*p* value	AN (*n* = 25)	Controls (*n* = 25)	*p* value
Total bone area (mm^2^)	251.9 ± 51.9	262.9 ± 47.8	0.56	680.7 ± 166.8	669.4 ± 119.2	0.78
Mean perimeter (mm)	66.2 ± 7.2	67.3 ± 6.2	0.56	101.0 ± 11.5	100.5 ± 8.7	0.86
Cortical area (mm^2^)	46.0 ± 11.6	52.8 ± 11.7	0.06	92.4 ± 21.5	123.1 ± 20.7	<**0.0001**
Trabecular area (mm^2^)	203.7 ± 54.9	206.5 ± 49.7	0.86	583.5 ± 177.9	545.2 ± 122.1	0.38
Cortical thickness (mm)	0.71 ± 0.23	0.79 ± 0.20	0.20	0.94 ± 0.29	1.24 ± 0.24	<**0.0005**
vBMD						
Total vBMD (mg/cm^3^)	284.7 (229.6; 312.9)	298.8 (280.0; 340.1)	0.10	251.3 ± 72.5	320.1 ± 48.7	<**0.0005**
Cortical vBMD (mg/cm^3^)	861. ± 78.9	878.4 ± 56.6	0.17	882.5 (866.2; 901.8)	912.2 (903.6; 925.1)	<**0.01**
Trabecular vBMD, mg/cm^3^	124.8 ± 35.9	155.7 ± 34.9	<**0.005**	137.1 ± 38.3	178.7 ± 31.0	<**0.0005**
Microarchitecture						
BV/TV, [1]	0.104 ± 0.030	0.130 ± 0.029	<**0.005**	0.114 ± 0.032	0.150 ± 0.025	<**0.0001**
Tb.Th. (mm)	0.063 ± 0.012	0.067 ± 0.012	0.18	0.069 ± 0.012	0.078 ± 0.013	<**0.01**
Tb.N (1/mm)	1.64 ± 0.27	1.92 ± 0.29	<**0.001**	1.64 ± 0.26	1.93 ± 0.30	<**0.001**
Tb.Sp (mm)	0.563 (0.468; 0.625)	0.432 (0.400; 0.490)	<**0.001**	0.554 ± 0.102	0.4512 ± 0.079	<**0.0005**
Cortical porosity (%)	0.72 (0.56; 1.15)	0.96 (0.63; 1.09)	0.55	2.65 (1.90; 3.59)	2.71 (2.21; 3.36)	0.70
Estimated strength						
Stiffness	61,444 ± 12,520	74,868 ± 13,673	<**0.001**	162,158 ± 27,161	214,192 ± 33,105	<**0.0001**
Failure load (N)	3115 ± 612	3810 ± 675	<**0.0005**	8242 ± 1284	10,727 ± 1624	<**0.0001**

Values are presented as mean ± SD or median and interquartile range, as appropriate. *p* values in bold indicates *p* < 0.05 on difference between groups

*AN* anorexia nervosa, *vBMD* volumetric bone mineral density, *BV/TV*. bone volume/tissue volume, *Tb.Th*. trabecular thickness, *Tb.N*. trabecular number, *Tb.Sp*. trabecular spacing

#### Distal Radius

**Fig. 1:**
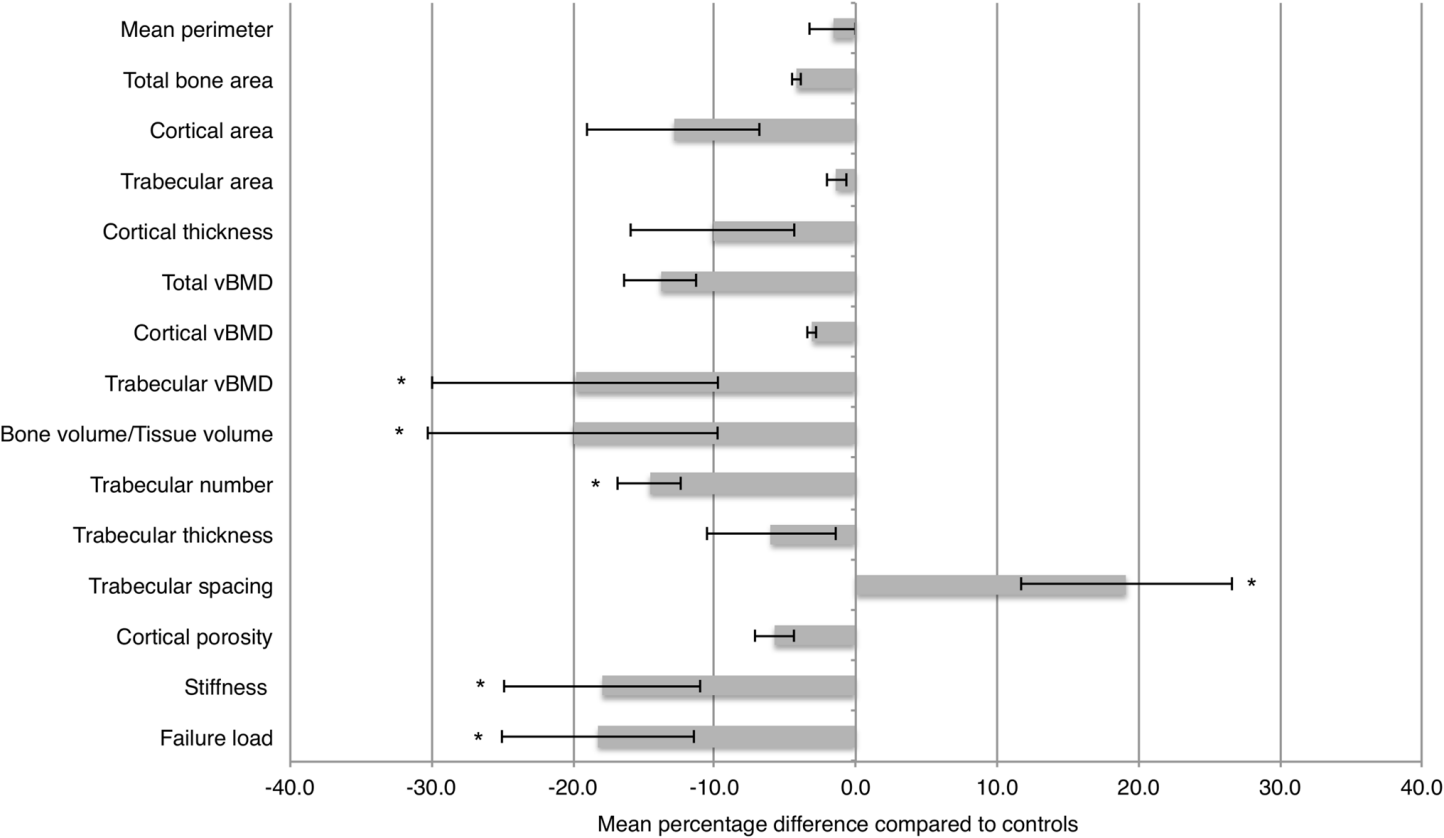
Mean percentage difference between patients and controls of measured geometric, volumetric and microarchitectural values in radius. *Error bars* indicate mean ± one standard deviation. *p value < 0.05

In the non-weight-bearing radius, patients and controls did not differ in the geometric indices of outer bone perimeter, total bone area, cortical area, trabecular area or cortical thickness (Fig. 1). Trabecular vBMD was lower in patients compared to controls (*p* < 0.005), but no differences were found in cortical vBMD or total vBMD. In terms of microarchitecture, BV/TV was significantly lower in the patient group (*p* < 0.005), due to a lower Tb.N. (*p* < 0.001), but there was no difference in Tb.Th. Accordingly, Tb.Sp. was higher in the patient group (*p* < 0.001). Cortical porosity was comparable between groups. Estimates of the overall mechanical properties of the bone (stiffness and failure load) were markedly lower in the AN group, compared to controls (*p* < 0.001 and *p* < 0.0005, respectively).

#### Distal Tibia

**Fig. 2:**
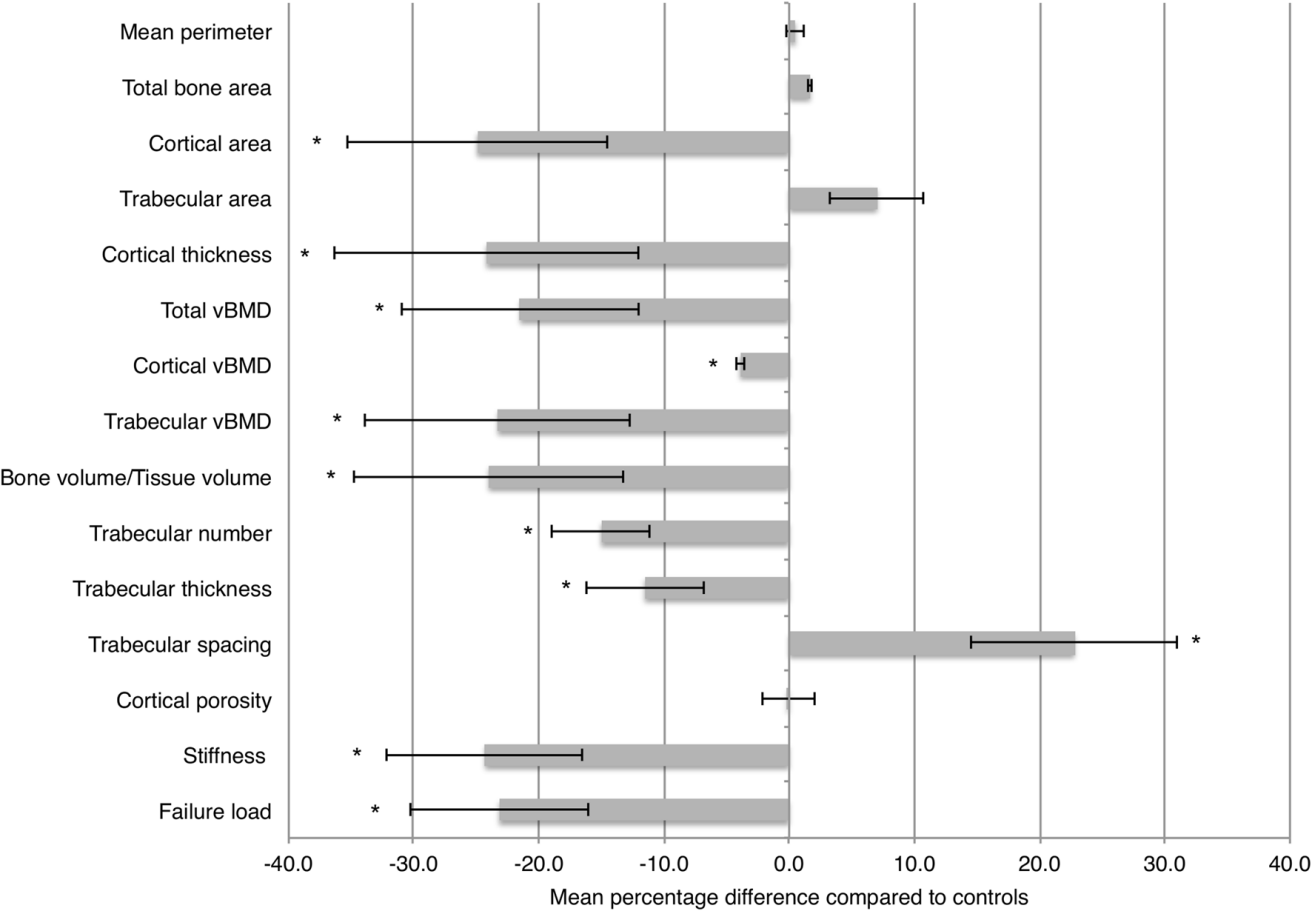
Mean percentage difference between patients and controls of measured geometric, volumetric and microarchitectural values in tibia. *Error bars* indicate mean ± one standard deviation. *p value < 0.05

In the weight-bearing tibia, outer bone perimeter and total bone area were comparable between groups. The cortex was significantly thinner in the patient group (*p* < 0.0005), the cortical area lower (*p* < 0.0001) and the trabecular area increased compared to controls, although the latter did not reach statistical significance (Fig. 2). Volumetric BMD was significantly lower in both the cortical and trabecular departments, as well as total vBMD (*p* < 0.01; *p* < 0.0005; *p* < 0.0005, respectively). In terms of trabecular microarchitecture, both trabecular number (*p* < 0.001), trabecular thickness (*p* < 0.01) and BV/TV (<0.0001) were lower in patients compared to controls, while trabecular spacing was higher in the patient group (*p* < 0.0005). Last, both stiffness and estimated failure load were markedly lower in the patient group (*p* < 0.0001 for both).

### Sensitivity Analyses

There were no significant association between OCP use among patients with AN, and any parameter obtained from the HR-pQCT scans (Supplemental Table 1).

### Regression Analysis

In the radius, decreasing weight in the patient group was associated with a decreasing Tb.N. and increasing Tb.Sp. (Table 4). For all other measures, there were no significant association to decreasing weight. In the tibia, decreasing patient weight was associated with a decrease in cortical thickness, Tb.N. and an increase in Tb.Sp. (Table 4). Both estimated measures of bone strength, stiffness and failure load decreased with decreasing weight.

**Table 4:** Regressions on HR-pQCT-derived measures and body weight. Estimates reflects the predicted change, when the weight of the patients, compared to controls, decreases with one kg

	Radius		Tibia		Radius versus tibia
	Estimate	*p* value	Estimate	*p* value	*p* value
Total vBMD (mg/cm^3^)	−1.31	0.40	−2.29	0.12	0.30
Trabecular vBMD (mg/cm^3^)	−1.11	0.23	−0.72	0.45	0.51
Cortical vBMD (mg/cm^3^)	−0.84	0.65	−1.64	0.16	0.70
Cortical thickness, mm	−0.003	0.62	−0.012	<**0.05**	<**0.05**
BV/TV	−0.0009	0.24	−0.0004	0.61	0.59
Tb.N. (1/mm)	−0.02	<**0.05**	−0.02	<**0.01**	0.81
Tb.Th. (mm)	0.0002	0.54	0.0004	0.08	0.52
Tb.Sp. (mm)	0.006	<**0.05**	0.006	<**0.05**	0.97
Stiffness	−394	0.25	−1720	<**0.05**	<**0.05**
Failure load (N)	−19.0	0.26	−90.8	<**0.05**	<**0.01**

Radius versus tibia reflects comparison of β-coefficients from the radius and tibia regressions. Values are adjusted for smoking. *p* values in bold indicates *p* < 0.05

*vBMD* volumetric bone mineral density, *BV/TV* bone volume/tissue volume, *Tb.Th*. trabecular thickness, *Tb.N*. trabecular number, *Tb.Sp*. trabecular spacing

When comparing the non-weight-bearing radius to the weight-bearing tibia, the regression coefficient differed significantly for cortical thickness, stiffness and estimated failure load.

## Discussion

In this cross-sectional study, we demonstrated differences in impairment between weight- and non-weight-bearing bones in AN, compared to normal weight controls. Key findings include (1) similar bone size between patients and controls, (2) a significant difference in cortical thickness in the tibia, but not in the radius and (3) a different association between body weight and both cortical thickness and estimated failure load in weight-bearing versus non-weight-bearing bone.

In the inclusion, we allowed for the use of OCP in both patient and control groups. The decision was based on the systemic review and meta-analyses by Sim et al. which failed to show a convincing effect of OCPs on BMD [24], and to the best of our knowledge, no effect of OCPs on bone geometry or microarchitecture has been shown. As OCPs are widely used in clinical populations of AN, exclusion of OCP users could be a threat to the external validity of the study. Reassuringly, sensitivity analyses on our data showed no independent effect of OCP use on any of the parameters derived from the HR-pQCT scans (Supplemental Table 1).

The finding of comparable bone perimeter and total bone area between patients and controls in both the radius and the tibia holds important implications. During growth, periosteal apposition exceeds endocortical resorption, mediating expansion of the bone and an increase in cortical thickness [25]. If the process of periosteal deposition was significantly impaired as a consequence of malnutrition and low weight in AN, a smaller bone perimeter was expected in the AN group, with the effect being most pronounced in patients with debut of disease at an early age. This, however, was not the case, nor when stratifying for age at onset of disease (data not shown). Our findings are in line with other HR-pQCT studies [26, 27] comparing patients with AN to normal weight controls, suggesting that decreased periosteal apposition is not the cause of bone impairment in AN.

In our study, cortical thickness was lower in the AN group in the weight-bearing tibia, but not in the non-weight-bearing radius (Fig. 1). Previous studies have shown significant cortical thinning in the radius as well [4, 26], possible due to an earlier onset of disease, compared to participants in our sample. In support of our results, at least one other study has shown that the magnitude of cortical thinning is higher in the tibia compared to the radius [27]. Thus, it seems that the overall impact of AN on cortical bone geometry is on the endocortical surface, either as a result of decreased formation during growth, or as increased resorption later on, but also that there may be a site-specific difference.

This proposed pattern of impaired cortical bone geometry, seems at least in part to resemble that of female menopause, exemplified by Szulc et al. [28] and later by Shanbhogue et al. [29]. Based on longitudinal assessment of healthy subjects, both studies highlight cortical thinning due to endocortical resorption as a hallmark of bone loss in postmenopausal women, but not in men [29], suggesting a potential role of estrogen. A possible link between estrogen deficiency and cortical bone loss in weight-bearing vs. non-weight-bearing bones, have been proposed by Lee et al. [30]. In a study on mice comparing a mouse strain lacking the estrogen receptor alpha (ER-alpha), with a strain with functional ER-alpha receptors, the effect of repeated mechanical loading on cortical area was diminished threefold in the knockout strain. In accordance, although not in patients with AN, a Finnish study including 245 girls aged 10–13 years, showed that polymorphisms of the ER-alpha gene modulated the effect of loading on weight-bearing, but not in non-weight-bearing bone [31]. Thus, it is possible that the difference in cortical thickness between weight-bearing and non-weight-bearing bone in our study was, at least in part, due to lower mechanical load, and that the effect might have been modulated by the lack of estrogen.

We report impaired trabecular microarchitecture in both the radius and the tibia. The pattern of trabecular bone impairment is comparable between the weight-bearing and non-weight-bearing bone, with lower values for BV/TV and Tb.N. and higher Tb.Sp. Even though there was no significant difference in total vBMD between groups in the radius, patients had markedly lower FEA-derived estimated failure load in both the radius and the tibia. This apparent divergence highlights that trabecular microarchitecture adds to calculated bone strength, beyond BMD. In postmenopausal women, a recent multicentre study found trabecular microarchitecture to modestly improve the discrimination of women with and without fracture independently of aBMD at the hip and vBMD at the radius or tibia [32]. Similar studies are yet to be made in an AN population. As forearm fractures are twice as prevalent in AN compared to healthy controls of same age [9], focusing on vBMD alone would probably cause an underestimation of fracture risk in our sample of patients.

In the regression analyses, we assessed the association between body weight and HR-pQCT variables, taking advantage of patients and controls being matched on height. This association was obviously confounded by disease severity, as a large weight difference between patient and control would most likely mean that the patient was more affected by malnutrition, compared to a patient-control pair with a smaller weight difference. By comparing weight-bearing to non-weight-bearing bone in each patient-control pairs, we were able to minimize this confounding issue. We report a positive association between weight and the predicted cortical thickness in tibia, but not in the radius (Table 4). If the relationship between weight and cortical thickness were solely due to weight serving as a proxy for disease severity, we would not expect a difference between weight-bearing and non-weight-bearing bone. Thus, the reported difference might be explained by mechanical loading enforced by body weight. On the contrary, predicted Tb.N. did decrease with decreasing patient weight, but there were no difference between weight-bearing and non-weight-bearing bone, making a role of mechanical loading, less likely. Finally, we found no significant association of predicted estimated failure load to weight in the radius, but a significant association in the tibia, again suggesting a role of body weight on estimated bone strength.

So what is the clinical implication of the proposed role of mechanical loading on cortical bone geometry and bone strength in weight-bearing bone? Is it an irreversible consequence of the disease or a reversible adaptation to low weight? The mechanical strength of the weight-bearing bone is without doubt decreased in AN, but so is the biomechanical requirements due to the decreased weight. Only longitudinal studies can show how bone microarchitecture respond to nutritional rehabilitation in AN, and define the prognostic potential in predicting fracture compared to aBMD.

### Strength and Limitations

The methodological strengths of our study include matching tightly on height. When including subjects of different height (and thereby different length of the bones), the region of interest is not identical between subjects. Shanbhogue et al. recently highlighted the importance of this methodological issue [33]. In their study comparing fixed offset to an offset relative to bone length, they report morphologic variation with varying measurement position, up to as much as 34% in the radius and 36% in the tibia. As height is highly correlated to arm and leg length in subjects of the same race, gender and age, we ensured agreement on regions of interest between groups, thereby making the direct comparison reliable. Another important strength was the random recruitment of control subjects, making the sample more likely to reflect the general population.

Important limitations include the cross-sectional design of the study, which especially relates to the emphasis on body weight. We recorded body weight at the day of the examination, but clearly the patient’s bones are affected by weight and disease-severity over time. A dramatic weight-change (either way) just before the examination could alter the relationship between weight and the measured variables. Smoking turned out to be more prevalent in the AN group, which represents a potential issue, as smoking is associated to decreased BMD and increased fracture risk [34]. However, as we adjusted for smoking in the regression analyses, the estimates are not affected by this in-balance in smoking prevalence. Finally, we did not adjust the HR-pQCT measurements for multiple testing. As the direction and magnitude of the measured difference between patients and controls were both consistent and intuitive throughout our data, we consider the risk of making a type 1 error unlikely, although it cannot be fully dismissed.

### Conclusion

In this cross-sectional study, we found significantly lower bone mass and impaired bone microarchitecture in adult AN patients, compared to normal weight controls. Furthermore, we demonstrate differences in bone impairment between weight-bearing and non-weight-bearing bone, implying an effect of the lower mechanical loading on bone geometry and microarchitecture in AN. Longitudinal studies are needed to show whether these changes are a reversible adaption to decreased mechanical needs, or represent irreversible damage to the skeleton, with implication also for long-term fracture risk in these patients.

## Electronic supplementary material

Below is the link to the electronic supplementary material.

Supplementary material 1 (DOCX 151 KB)
