# Supplementary material for: The Role of Body Weight on Bone in Anorexia Nervosa: A HR-pQCT Study
Source: Calcif Tissue Int. 2017 Feb 21;101(1):24–33. doi: 10.1007/s00223-017-0254-7 (PMC5486942; doi:10.1007/s00223-017-0254-7)
Supplement: Supplementary file 1 — Supplementary material 1 (DOCX 151 KB) [file 223_2017_254_MOESM1_ESM.docx]

**Supplemental tables**

|  | Radius | | Tibia | |
| --- | --- | --- | --- | --- |
|  | Coefficient (95% CI) | P-value | Coefficient (95% CI) | P-value |
| Geometry |  |  |  |  |
| Total bone area mm2 | 10.1 (-27.5; 47.6) | 0.58 | 1.92 (-96.15; 99.99) | 0.97 |
| Mean perimeter, mm | 1.12 (-4.6; 6.9) | 0.69 | 0.12 (-7.25; 7.48) | 0.97 |
| Cortical area, mm2 | 7.1 (-1.0; 15.2) | 0.08 | 7.8 (-9.8; 25.2) | 0.37 |
| Trabecular area, mm2 | 3.2 (-36.3; 42.6) | 0.86 | -4.0 (-111.0; 103.1) | 0.94 |
| Cortical thickness, mm | 0.11 (-0.05; 0.27) | 0.18 | 0.09 (-0.14; 0.32) | 0.44 |
| vBMD |  |  |  |  |
| Total vBMD, mg/cm3 | 40.1 (-25.2; 105.3) | 0.22 | 21.5 (-37.6; 80.5) | 0.46 |
| Cortical vBMD, mg/cm3 | 21.8 (-27.3; 70.9) | 0.37 | 24.4 (-16.7; 65.5) | 0.23 |
| Trabecular vBMD, mg/cm3 | 13.6 (-19.7; 46.9) | 0.40 | 6.7 (-27.9; 43.2) | 0.66 |
| Microarchitecture |  |  |  |  |
| BV/TV, [1] | 0.012 (-0.016; 0.039) | 0.39 | 0.006 (-0.023; 0.036) | 0.66 |
| Tb.Th., mm | 0.008 (-0.004; 0.019) | 0.18 | -0.0002 (-0.0106; 0.0102) | 0.97 |
| Tb.N, 1/mm | -0.025 (-0.292; 0.242) | 0.85 | 0.087 (-0.166; 0.339) | 0.48 |
| Tb.Sp, mm | 0.002 (-0.105; 0.110) | 0.96 | -0.023 (-0.123; 0.075) | 0.62 |
| Cortical porosity, % | -0.003 (-0.009; 0.002) | 0.26 | -0.012 (-0.024; 0.0001) | 0.06 |
| Estimated strength |  |  |  |  |
| Stiffness | 9169 (-364; 18702) | 0.07 | 5252 (-19524; 30028) | 0.66 |
| Failure load, N | 431 (-50; 912) | 0.08 | 289 (-901; 1480) | 0.62 |

**Supplemental table 1** The association between use of oral contraception pills (OCP) and HR-pQCT parameters, adjusted for duration of disease and BMI. Values are presented as regression coefficients followed by 95 % confidence intervals (CI). P-values in bold indicates p<0.05.

| Geometry | AN (n=25) | AN, no OCP use (n=16) | Controls (n=25) | P-value, all patients vs. controls | P-value, no OCP use vs. controls |
| --- | --- | --- | --- | --- | --- |
| Total bone area mm2 | 251.9 ± 51.9 | 260.6 +/- 50.8 | 262.9 ± 47.8 | 0.56 | 0.89 |
| Mean perimeter, mm | 66.2 ± 7.2 | 66.9 +/-7.3 | 67.3 ± 6.2 | 0.56 | 0.86 |
| Cortical area, mm2 | 46.0 ± 11.6 | 43.6 +/- 9.2 | 52.8 ± 11.7 | 0.06 | **<0.05** |
| Trabecular area, mm2 | 203.7 ± 54.9 | 208.6 +/- 53.9 | 206.5 ± 49.7 | 0.86 | 0.71 |
| Cortical thickness, mm | 0.71 ± 0.23 | 0.68 +/- 0.19 | 0.79 ± 0.20 | 0.2 | 0.08 |
| vBMD |  |  |  |  |  |
| Total vBMD, mg/cm3 | 284.7 (229.6; 312.9) | 272.9 (213.7; 309.15) | 298.8 (280.0; 340.1) | 0.1 | 0.06 |
| Cortical vBMD, mg/cm3 | 861.0 ± 78.9 | 841 +/-82.6 | 878.4 ± 56.6 | 0.17 | **<0.05** |
| Trabecular vBMD, mg/cm3 | 124.8 ± 35.9 | 118.2 +/- 31.2 | 155.7 ± 34.9 | **<0.005** | **<0.005** |
| Microarchitecture |  |  |  |  |  |
| BV/TV, [1] | 0.104 ± 0.030 | 0.098 +/- 0.026 | 0.130 ± 0.029 | **<0.005** | **<0.001** |
| Tb.Th., mm | 0.063 ± 0.012 | 0.060 +/- 0.011 | 0.067 ± 0.012 | 0.18 | **<0.05** |
| Tb.N, 1/mm | 1.64 ± 0.27 | 1.64 +/- 0.27 | 1.92 ± 0.29 | **<0.001** | **<0.005** |
| Tb.Sp, mm | 0.563 (0.468; 0.625) | 0.558 (0.491; 0.626 | 0.432 (0.400; 0.490) | **<0.001** | **<0.005** |
| Cortical porosity, % | 0.72 (0.56; 1.15) | 0.76 (5.9; 13.5) | 0.96 (0.63; 1.09) | 0.55 | 0.16 |
| Estimated strength |  |  |  |  |  |
| Stiffness | 61444 ± 12520 | 57527 +/- 9426 | 74868 ± 13673 | **<0.001** | **<0.0001** |
| Failure load, N | 3115 ± 612 | 2975.13 +/- 464 | 3810 ± 675 | **<0.0005** | **<0.0001** |

**Supplemental table 2** Radius HR-pQCT measures in all patients vs. controls and the subgroup of patients not using oral contraception pills (OCP) vs. controls. Values are presented as mean ± SD or median and interquartile range, as appropriate. P-values in bold indicates p<0.05 on difference between groups. AN = anorexia nervosa; vBMD = volumetric bone mineral density; BV/TV = bone volume/tissue volume; Tb.Th. = trabecular thickness; Tb.N. = trabecular number; Tb.Sp. = trabecular spacing

| Geometry | AN (n=25) | AN, no OCP use (n=16) | Controls (n=25) | P-value, all patients vs. controls | P-value, no OCP use vs. controls |
| --- | --- | --- | --- | --- | --- |
| Total bone area mm2 | 680.7 ± 166.8 | 706.4 +/- 177.9 | 669.4 ± 119.2 | 0.78 | 0.43 |
| Mean perimeter, mm | 101.0 ± 11.5 | 102.7 +/- 12.0 | 100.5 ± 8.7 | 0.86 | 0.49 |
| Cortical area, mm2 | 92.4 ± 21.5 | 87.3 +/- 22.3 | 123.1 ± 20.7 | **<0.0001** | **<0.0001** |
| Trabecular area, mm2 | 583.5 ± 177.9 | 613.1 +/- 187.9 | 545.2 ± 122.1 | 0.38 | 0.17 |
| Cortical thickness, mm | 0.94 ± 0.29 | 0.875 +/- 0.281 | 1.24 ± 0.24 | **<0.0005** | **<0.0001** |
| vBMD |  |  |  |  |  |
| Total vBMD, mg/cm3 | 251.3 ± 72.5 | 234.7 +/- 62.8 | 320.1 ± 48.7 | **<0.0005** | **<0.0001** |
| Cortical vBMD, mg/cm3 | 882.5 (866.2; 901.8) | 869.1 (863.9; 891.9) | 912.2 (903.6; 925.1) | **<0.01** | **<0.005** |
| Trabecular vBMD, mg/cm3 | 137.1 ± 38.3 | 131.1 +/- 36.0 | 178.7 ± 31.0 | **<0.0005** | **<0.0001** |
| Microarchitecture |  |  |  |  |  |
| BV/TV, [1] | 0.114 ± 0.032 | 0.109 +/- 0.029 | 0.150 ± 0.025 | **<0.0001** | **<0.0001** |
| Tb.Th., mm | 0.069 ± 0.012 | 0.067 +/- 0.012 | 0.078 ± 0.013 | **<0.01** | **<0.05** |
| Tb.N, 1/mm | 1.64 ± 0.26 | 1.61 +/- 0.22 | 1.93 ± 0.30 | **<0.001** | **<0.001** |
| Tb.Sp, mm | 0.554 ± 0.102 | 0.567 +/- 0.096 | 0.4512 ± 0.079 | **<0.0005** | **<0.0005** |
| Cortical porosity, % | 2.65 (1.90; 3.59) | 3.4 (2.1; 4.2) | 2.71 (2.21; 3.36) | 0.7 | 0.65 |
| Estimated strength |  |  |  |  |  |
| Stiffness | 162158 ± 27161 | 157519 +/- 28235 | 214192 ± 33105 | **<0.0001** | **<0.0001** |
| Failure load, N | 8242 ± 1284 | 8029 +/- 1335 | 10727 ± 1624 | **<0.0001** | **<0.0001** |

**Supplemental table 3** Tibia HR-pQCT measures in all patients vs. controls and the subgroup of patients not using oral contraception pills (OCP) vs. controls. Values are presented as mean ± SD or median and interquartile range, as appropriate. P-values in bold indicates p<0.05 on difference between groups. AN = anorexia nervosa; vBMD = volumetric bone mineral density; BV/TV = bone volume/tissue volume; Tb.Th. = trabecular thickness; Tb.N. = trabecular number; Tb.Sp. = trabecular spacing

| Geometry | AN (n=25) | AN, non smokers (n=16) | Controls (n=25) | P-value, all patients vs. controls | P-value, non smokers vs. controls |
| --- | --- | --- | --- | --- | --- |
| Total bone area mm2 | 251.9 ± 51.9 | 254.6 ± 62.7 | 262.9 ± 47.8 | 0.56 | 0.65 |
| Mean perimeter, mm | 66.2 ± 7.2 | 66.0 ± 8.6 | 67.3 ± 6.2 | 0.56 | 0.58 |
| Cortical area, mm2 | 46.0 ± 11.6 | 46.2 ± 14.4 | 52.8 ± 11.7 | 0.06 | 0.13 |
| Trabecular area, mm2 | 203.7 ± 54.9 | 202.9 ± 67.8 | 206.5 ± 49.7 | 0.86 | 0.85 |
| Cortical thickness, mm | 0.71 ± 0.23 | 0.73 ± 0.29 | 0.79 ± 0.20 | 0.2 | 0.41 |
| vBMD |  |  |  |  |  |
| Total vBMD, mg/cm3 | 284.7 (229.6; 312.9) | 291.9 (269.8; 312.9) | 298.8 (280.0; 340.1) | 0.1 | 0.46 |
| Cortical vBMD, mg/cm3 | 861.0 ± 78.9 | 855.9 ± 98.5 | 878.4 ± 56.6 | 0.17 | 0.20 |
| Trabecular vBMD, mg/cm3 | 124.8 ± 35.9 | 129.5 ± 34.6 | 155.7 ± 34.9 | **<0.005** | **<0.05** |
| Microarchitecture |  |  |  |  |  |
| BV/TV, [1] | 0.104 ± 0.030 | 0.108 ± 0.030 | 0.130 ± 0.029 | **<0.005** | **<0.05** |
| Tb.Th., mm | 0.063 ± 0.012 | 0.066 ± 0.011 | 0.067 ± 0.012 | 0.18 | 0.76 |
| Tb.N, 1/mm | 1.64 ± 0.27 | 1.68 ± 0.27 | 1.92 ± 0.29 | **<0.001** | **<0.01** |
| Tb.Sp, mm | 0.563 (0.468; 0.625) | 0.553 (0.446; 0.589) | 0.432 (0.400; 0.490) | **<0.001** | **<0.05** |
| Cortical porosity, % | 0.72 (0.56; 1.15) | 0.75 (0.57; 1.2) | 0.96 (0.63; 1.09) | 0.55 | 0.62 |
| Estimated strength |  |  |  |  |  |
| Stiffness | 61444 ± 12520 | 62850 ± 14617 | 74868 ± 13673 | **<0.001** | **<0.005** |
| Failure load, N | 3115 ± 612 | 3194 ± 705 | 3810 ± 675 | **<0.0005** | **<0.005** |

**Supplemental table 4** Radius HR-pQCT measures in all patients vs. controls and the subgroup of patients who do not smoke vs. controls. Values are presented as mean ± SD or median and interquartile range, as appropriate. P-values in bold indicates p<0.05 on difference between groups. AN = anorexia nervosa; vBMD = volumetric bone mineral density; BV/TV = bone volume/tissue volume; Tb.Th. = trabecular thickness; Tb.N. = trabecular number; Tb.Sp. = trabecular spacing

| Geometry | AN (n=25) | AN, non-smokers (n=13) | Controls (n=25) | P-value, all patients vs. controls | P-value, non smokers vs. controls |
| --- | --- | --- | --- | --- | --- |
| Total bone area mm2 | 680.7 ± 166.8 | 661.4 ± 126.6 | 669.4 ± 119.2 | 0.78 | 0.85 |
| Mean perimeter, mm | 101.0 ± 11.5 | 99.9 ± 9.3 | 100.5 ± 8.7 | 0.86 | 0.86 |
| Cortical area, mm2 | 92.4 ± 21.5 | 91.2 ± 22.1 | 123.1 ± 20.7 | **<0.0001** | **<0.0001** |
| Trabecular area, mm2 | 583.5 ± 177.9 | 566.2 ± 139.6 | 545.2 ± 122.1 | 0.38 | 0.64 |
| Cortical thickness, mm | 0.94 ± 0.29 | 0.93 ± 0.29 | 1.24 ± 0.24 | **<0.0005** | **<0.005** |
| vBMD |  |  |  |  |  |
| Total vBMD, mg/cm3 | 251.3 ± 72.5 | 244.7 ± 83.3 | 320.1 ± 48.7 | **<0.0005** | **<0.005** |
| Cortical vBMD, mg/cm3 | 882.5 (866.2; 901.8) | 877.0 (864.8; 901.8) | 912.2 (903.6; 925.1) | **<0.01** | **<0.01** |
| Trabecular vBMD, mg/cm3 | 137.1 ± 38.3 | 142.7 ± 40.0 | 178.7 ± 31.0 | **<0.0005** | **<0.005** |
| Microarchitecture |  |  |  |  |  |
| BV/TV, [1] | 0.114 ± 0.032 | 0.115 ± 0.035 | 0.150 ± 0.025 | **<0.0001** | **<0.001** |
| Tb.Th., mm | 0.069 ± 0.012 | 0.069 ± 0.012 | 0.078 ± 0.013 | **<0.01** | **<0.05** |
| Tb.N, 1/mm | 1.64 ± 0.26 | 1.64 ± 0.30 | 1.93 ± 0.30 | **<0.001** | **<0.01** |
| Tb.Sp, mm | 0.554 ± 0.102 | 0.558 ± 0.116 | 0.4512 ± 0.079 | **<0.0005** | **<0.005** |
| Cortical porosity, % | 2.65 (1.90; 3.59) | 2.65 (2.21; 3.21) | 2.71 (2.21; 3.36) | 0.7 | 0.81 |
| Estimated strength |  |  |  |  |  |
| Stiffness | 162158 ± 27161 | 160655 ± 29158 | 214192 ± 33105 | **<0.0001** | **<0.0001** |
| Failure load, N | 8242 ± 1284 | 8186 ± 1392 | 10727 ± 1624 | **<0.0001** | **<0.0001** |

**Supplemental table 5** Tibia HR-pQCT measures in all patients vs. controls and the subgroup of patients who do not smoke vs. controls. Values are presented as mean ± SD or median and interquartile range, as appropriate. P-values in bold indicates p<0.05 on difference between groups. AN = anorexia nervosa; vBMD = volumetric bone mineral density; BV/TV = bone volume/tissue volume; Tb.Th. = trabecular thickness; Tb.N. = trabecular number; Tb.Sp. = trabecular spacing
